# Vectorized nanodelivery systems for ischemic stroke: a concept and a need

**DOI:** 10.1186/s12951-017-0264-7

**Published:** 2017-04-11

**Authors:** Andrés Da Silva-Candal, Bárbara Argibay, Ramón Iglesias-Rey, Zulema Vargas, Alba Vieites-Prado, Esteban López-Arias, Emilio Rodríguez-Castro, Iria López-Dequidt, Manuel Rodríguez-Yáñez, Yolanda Piñeiro, Tomás Sobrino, Francisco Campos, José Rivas, José Castillo

**Affiliations:** 1 Department of Neurology, Clinical Neurosciences Research Laboratory, Hospital Clínico Universitario, Universidade de Santiago de Compostela, Health Research Institute of Santiago de Compostela (IDIS), c/Travesa da Choupana, s/n, 15706 Santiago de Compostela, Spain; 2 Nanomag Laboratory, Department of Applied Physics, Technological Research Institute, Universidade de Santiago de Compostela, Health Research Institute of Santiago de Compostela (IDIS), Campus Vida, 15782 Santiago de Compostela, Spain

**Keywords:** Human disease, Ischemic stroke, Nanoparticles, Nanotechnology, Specific targeting

## Abstract

Neurological diseases of diverse aetiologies have significant effects on the quality of life of patients. The limited self-repairing capacity of the brain is considered to be the origin of the irreversible and progressive nature of many neurological diseases. Therefore, neuroprotection is an important goal shared by many clinical neurologists and neuroscientists. In this review, we discuss the main obstacles that have prevented the implementation of experimental neuroprotective strategies in humans and propose alternative avenues for the use of neuroprotection as a feasible therapeutic approach. Special attention is devoted to nanotechnology, which is a new approach for developing highly specific and localized biomedical solutions for the study of the multiple mechanisms involved in stroke. Nanotechnology is contributing to personalized neuroprotection by allowing us to identify mechanisms, determine optimal therapeutic windows, and protect patients from brain damage. In summary, multiple aspects of these new *players* in biomedicine should be considered in future in vivo and in vitro studies with the aim of improving their applicability to clinical studies.

## Background

The socioeconomic repercussions of neurological diseases are substantial. According to a 2010 study from the European Brain Council, the annual cost of neurological disease reaches 800 billion Euros per year, 60% of which is attributed to direct costs [1, 2]. In Europe, more than 8 million people have a stroke every year, with an associate cost of about 64 billion Euros. Considering that the ageing European population will rise up continuously, the clinical repercussions of neurological disease will also have large impacts on our health quality. Alterations of the nervous system that directly lead to motor, sensory, cognitive, and behavioural changes affect the character of the patient.

The limited self-repairing capacity of the brain is considered to be the origin of the irreversible and progressive nature of many neurological diseases. Therefore, neuroprotection may be thought of as a set of interventions used to improve the resilience of the nervous system, which is an important goal shared by many clinical neurologists and neuroscientists. Research on new drugs designed to block the progression of damage in the brain parenchyma began more than five decades ago, and the first clinical trials were initiated in the 1980s [3–5]. These studies have become one of the main sources of knowledge required to understand the nervous system. However, all of the early approaches failed when they were translated in clinical trials. This raised doubts regarding the applicability of human neuroprotection strategies [6, 7]. In fact, this unsuccessful studies had caused pharmaceutical companies to stop their research projects in neurology [8], although, nowadays scientific evidence allows us to ensure that neuroprotection (defined as a combination of strategies, therapies and drugs that may result in salvage, recovery or regeneration of the tissue loss, its cells, structure and function) is definitely feasible irrespective of the difficulties in finding the optimal conditions for clinical applications [9, 10].

In this paper, we review the main obstacles that have prevented the implementation of experimental neuroprotective therapeutics in humans and propose alternative avenues to bring back neuroprotection as a feasible therapeutic approach. Special attention will be devoted to nanotechnology as a new approach used to develop highly specific and localized biomedical solutions for multiple mechanisms involved in stroke. Nanostructures have been shown to be powerful and innovative tools in materials science, electronics, energy, and environmental science. Recently, nanostructures have been shown to have unexpected utility in biomedical applications, both for imaging and therapeutic development.

## Broadening the concept of neuroprotection

In the short period of time immediately after the reduction in cerebral blood flow in a particular brain region (from seconds to minutes), there are a series of sequential processes, such as biochemical, metabolic, and cellular alterations that eventually lead to tissue necrosis. This necrotic area, denominated core, will be surrounded by damaged, but potentially recoverable tissue, called ischemic penumbra [11].

Traditionally, neuroprotective studies have focused on protecting cells from damage by identifying drugs that are capable of blocking one or more components of the neuronal ischemic cascade in the penumbra [12, 13]. Of course, these concepts must be reviewed. Cellular death cascade begins in the ischemic penumbra area and affects neurons, other brain cells like astrocytes, oligodendrocytes, microglia and vascular cells like endothelial cells, pericytes, and smooth muscle cells. In addition, this cascade interferes with the interactions between brain cells and the extracellular matrix. Thus, the identification of neuroprotective targets for the treatment of cerebral ischemia must extend from the exclusive and limited neuronal damage that is observed, to the broad recovery of all neurovascular networks [14].

The evolution of the penumbra to viable tissue or to progressive destruction is conditioned by biochemical mechanisms triggered by cerebral ischemia, which in turn critically depends on excitotoxicity, oxidative stress, and inflammation. These pathophysiological responses affect not only neurons, but also the entire neurovascular system. Their impact and intensity vary over time, even during the acute phase of cerebral ischemia. These responses may lead to further destruction or protection, even within the same cell line [14–19].

Glutamate acts not only as a neurotransmitter, but also as a signalling system between the different cell types of the nervous system. The high level of extracellular glutamatergic excitotoxicity [20] in cerebral ischemia is due to increased glutamate release by neurons [21], the failure of astrocytic glutamate uptake, and disruption of the glutamate-glutamine cycle [22]. Moreover, since oligodendrocytes express *N*-methyl-d-aspartate (NMDA) and α-amino-3-hydroxy-5-methyl-4-isoxazolepropionic acid (AMPA) receptors, their associated toxicity blocks signalling between myelin and axons, contributing to neuronal injury [23]. Pericytes [24] and endothelial cells [25] also have glutamate receptors and are susceptible to excitotoxic damage. One possible reason for the clinical failure of glutamate antagonists may be their focus on neuronal NMDA receptors [26]. It may be that a more universal approach to reducing the concentrations of extracellular glutamate through non-specific receptor mechanisms would be a more promising alternative [27].

Oxidative stress increases mitochondrial permeability, causes oedema and mitochondrial damage, and ultimately leads to neuronal death. Free radicals are produced in large amounts in ischemic tissue, especially after reperfusion. This occurs both when the reperfusion is spontaneous and when it is induced as a part of a therapeutic approach. The astrocytes in turn release antioxidants that help neuronal survival [28].

Endothelial cells are an important source of nitric oxide. This compound, under normal conditions, promotes increases in blood flow. However, in the presence of other radicals, nitric oxide can be converted into peroxynitrite, which is capable of destroying cellular membranes [29]. Cerebral white matter, which has high lipid content, is another important source of free radicals, and contributes to oxidative damage of oligodendrocytes around neuronal axons. In addition, oxidative stress prevents from myelinogenesis and post-ischemia axonal recovery [30].

The inflammatory response is characterised by the accumulation of cells and inflammatory mediators in the ischemic brain and is responsible for endothelial damage and blood brain barrier (BBB) breakdown in the early course of ischemia [31]. This disruption facilitates the recruitment of inflammatory cells, the microglia activation, and neuronal destruction [32]. Microglia and other inflammatory cells in the brain express toll-like receptors, which are able to recognize damage-associated molecular pattern molecules (DAMPs), which induce the expression of inflammatory mediators, adhesion molecules and the activation of innate immunity [33, 34]. Reactive astrocytes also increase the production of pro-inflammatory cytokines blocking axonal recovery. Pericytes are also inflammatory mediators that, at the same time, induce further DAMP release [19, 35].

Exosomes are nanovesicles (40–100 nm) excreted by all brain cells. They contain lipids, proteins, and nucleic acids, which are essential for communicating between non-contiguous cells [36]. They are incorporated into target cells, where they modify or reprogram cellular activity depending on their contents. During cellular ischemia, some inflammatory mediators stimulate exosome production by endothelial cells [37]. Astrocytes, oligodendrocytes, microglia, and neurons are connected via exosome release, which has as yet unknown functions in many cases. Oligodendrocytic exosomes release interferes with oligodendrocytic control of microglia and provokes microglial activation [38–40].

**Fig. 1:**
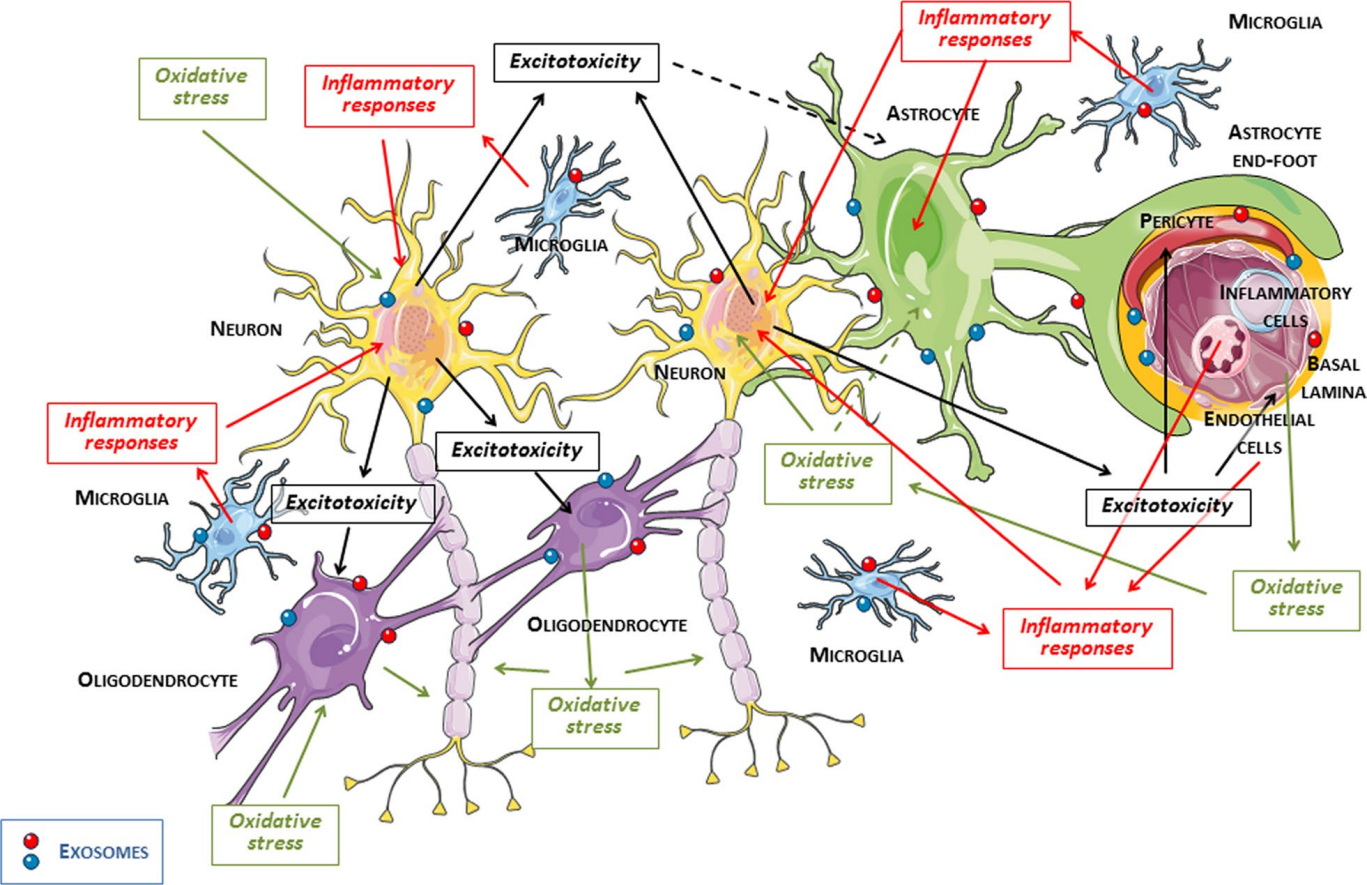
Cellular and intercellular complex of the neurovascular system and joint mechanisms of the ischemic cascade. *Continuous* and *dashed lines* indicate toxic and protective effects, respectively, during the acute phase of cerebral ischemia

Simplistic neuroprotective approaches that ignore the complex and interrelated cellular universe (Fig. 1) constituting the neurovascular system have little chance of success. Even though cell survival is necessary, it is not sufficient for complete structural and functional recovery after stroke. Thus, in addition to neuroprotection, it is crucial to ensure that the area surrounding the lesion has the appropriate conditions needed for restoration of cellular function in the affected region.

## Does neuroprotection still have a chance?

Intravenous thrombolysis administered within 4.5 h after the onset of ischemic stroke symptoms is probably the best therapeutic approach for patients at this time. Market pressure makes it difficult to compare this treatment to other non-pharmacological procedures, or even other alternative therapies. However, after 20 years of clinical experience and enormous economic investment, intravenous thrombolysis is applied only in 0.4–3% of cases in developing countries [41, 42], and in 2–7% of cases in most developed countries [43]. Revascularization treatment will certainly become more effective (and more expensive and difficult to sustain) in the future [44–46], but will probably not become a universal treatment [47].

Considering the above, it is necessary to insist on the development of neuroprotection as a treatment complementary to the at most 10% of patients who undergo revascularization treatment. Most importantly, neuroprotection should be developed as an alternative therapy for the 90% of patients that cannot be treated with revascularization therapy. An outcome improvement of 5 for 100% of patients will have more significant clinical and socioeconomic repercussions than an outcome improvement of 40 in 10% of the target population.

In preclinical studies, neuroprotection has been shown to be more effective in models of ischemia that are followed by artery reperfusion [5]. This has led to the postulate that neuroprotection may be appropriate only if cerebral perfusion is restored. However, if we restrict the use of neuroprotection to cases with restored perfusion, the added value of this approach to stroke treatment will be minimal and possibly limited to the inhibition of the oxidative stress effects after reperfusion [48]. Despite of findings that demonstrate the requisite close correlation between early recanalization and late clinical outcomes of direct relevance to the patient [49–53], the close relationships between recanalization, clinical benefit, and the pathophysiological changes secondary to reperfusion, have not yet entirely been elucidated [54, 55]. Firstly, in some cases there is no demonstrated association between recanalization frequency and clinical benefit (intra-arterial thrombolysis and mechanical thrombectomy lead to more recanalization, but both fail to improve clinical outcomes) [56–59]. Secondly, optimal collateral circulation may protect the brain parenchyma, even in the absence of recanalization [60]. Thirdly, early recanalization only confers clinical benefit to one-third of treated patients [61]. Fourthly, recanalization may exacerbate the tissue damage following hyperperfusion, cerebral oedema, and secondary haemorrhagic transformation [62]. Fifthly, reperfusion may not occur even when complete recanalization is achieved due to a few known mechanisms, which lead the phenomenon of “non-reflow” [63]. Finally, spontaneous recanalization is not an exceptional phenomenon, but occurs in 25% of patients during the first 24 h and in 50% of cases during the first week [55].

Therefore, neuroprotection remains a necessary opportunity for not only more than three-quarters of patients who will not benefit from a proper and safe recanalization treatment, but for all patients with ischemic stroke.

## The therapeutic window of neuroprotection

The establishment of therapeutic windows for different neuroprotective (or even recanalization) treatments is dependent on regulatory requirements, but does not always follow scientific evidence. The mantra “time is brain” has become an indisputable truth, but is not necessarily related to the pathophysiology of cerebral ischemia and it application may be different from one patient to another [64]. The administration of neuroprotective drugs, recanalization, or repair of injured tissue may be not mislead with the indisputable importance of a neurological diagnosis and specialized care as soon as possible in patients with suspected ischemic stroke.

Cerebral ischemia is a dynamic process during which time is not discontinuous, but is associated with continuous individualized hemodynamic, cellular, and molecular changes [65]. Therefore, not all therapeutic procedures may be indicated in all individuals at the same time. The onset of symptoms does not always coincide with the timing of vascular occlusion. Instead, a balance between the occlusion and suitable collateral circulation determines the onset of clinical signs. A progressive occlusion may allow the development of an intense collateral circulation that nullifies or minimizes neurological deficits. On the other hand, an acute occlusion does not facilitate collateral flow, and as a consequence, sharply damages the cerebral parenchyma [64].

**Fig. 2:**
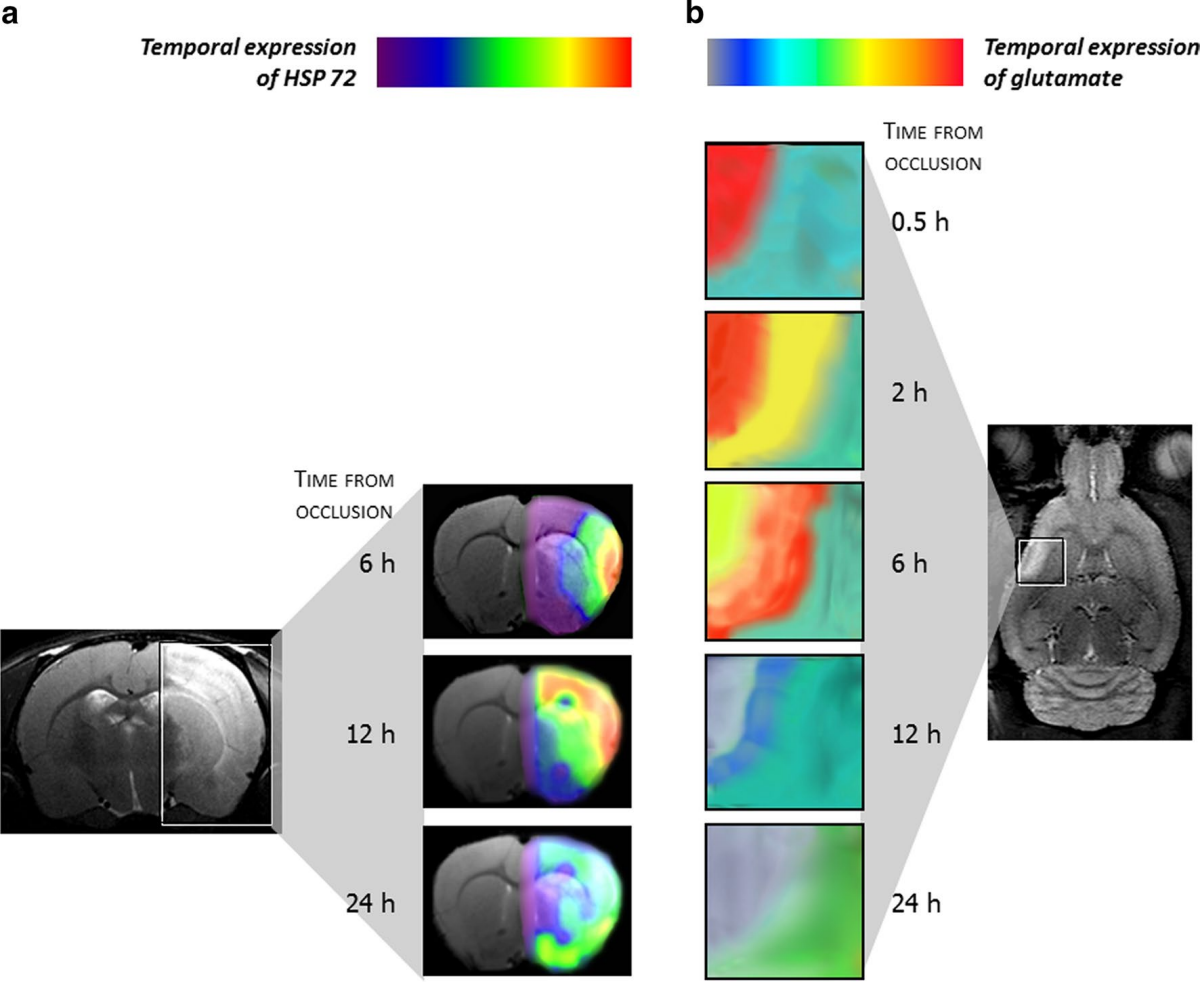
**a** Molecules expressed in a rat ischemia–reperfusion model. Temporal expression of heat shock protein-72 (colour-coded density maps overlaid on magnetic resonance images). The highest expression level is found in the cerebral cortex between 6 and 12 h after occlusion. **b** Temporal expression of glutamate (colour-coded spectroscopic maps). The maximum concentration occurs at 30 min following occlusion at the nucleus of the lesion, while the peak glutamate concentration in the periphery of the infarction occurs at 6 h after occlusion (Reproduced from [107] by permission of *Stroke*)

Recanalization treatment, although patient specific, is time-dependent [66], and has molecular and clinical markers, including those obtained using medical imaging, that allow the clinician to determine the best timing for individual treatment [67–69]. However, the therapeutic window for neuroprotective drugs may be different depending on whether the main goal is to control excitotoxicity, oxidative stress, or inflammation. The inhibition of post-ischemic inflammation in a very narrow therapeutic window has reduced effectiveness, similar to using glutamate grabbers many hours after the onset of symptoms (Fig. 2). In brief, the current challenge is recognize that the best therapeutic timing for a neuroprotective drug will be different depending on the affected brain area and treatment target.

## Dynamic and heterogenic ischemic pathophysiology

Recent studies have demonstrated that the components of the neurovascular unit have heterogenic characteristics, not only in morphology, function, gene expression, and physiological properties, but also in their responses to different diseases [70–72]. This heterogeneity may explain why these cells have developed harmful or protective responses at different times after cerebral ischemia [15].

**Fig. 3:**
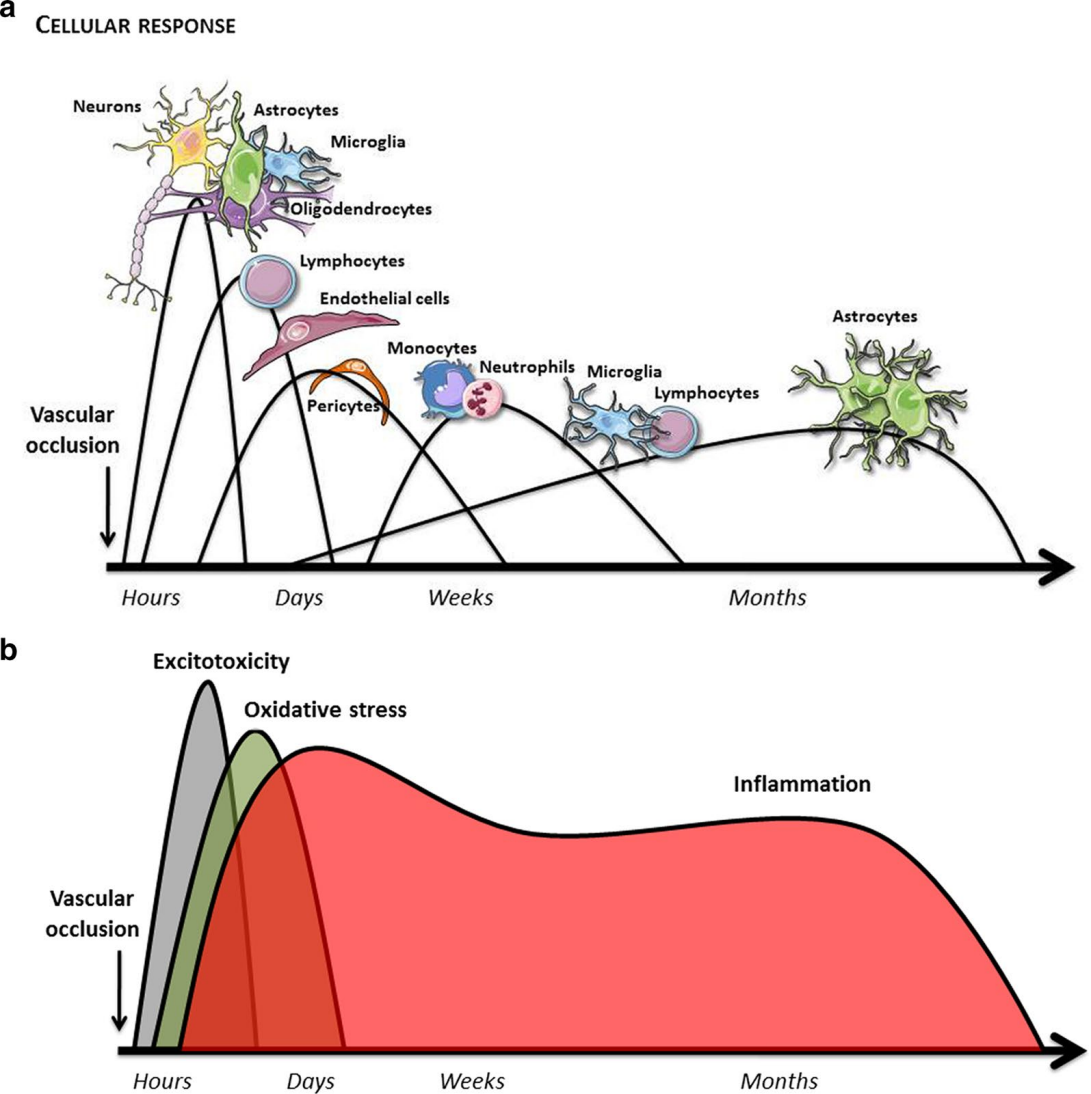
Time-courses of cellular (**a**) and molecular responses (**b**) during the chronological evolution of cerebral ischemia. Each of the cells involved and the secondary molecular expression patterns can play a bi- or tri-phasic role. These roles may sometimes be interrelated

The cellular and molecular responses that are observed during cerebral ischemia (Fig. 3) are sequential processes that progressively affect different cellular elements that may then determine downstream molecular responses, which are sometimes antagonistic. This possible limitation is one of the responsible of the development of the personalized medicine in this pathology. Therapy must be adapted to the cellular or process goal in function of time and the affected region. Once cerebral perfusion is reduced below the ischemic threshold, neurons initiate a calcium-dependent destructive process, and as a consequence release large amounts of glutamate and induce astrocytic reactivity. These reactivated astrocytes promote progressive tissue destruction by inhibiting glutamate uptake, releasing pro-inflammatory cytokines, and developing a glial scar, which will prevent ischemic injury repair. However, in a complementary manner, the stimulated astrocytes activate mechanisms that increase cerebral blood flow, stimulate genes needed for regulating neuronal synaptogenesis or the secretion of trophic factors, and contribute to BBB repair and myelinogenesis [70–75].

The breakdown of the blood brain barrier, which is secondary to endothelial dysfunction, is another key component in ischemic brain damage [76]. Nevertheless, damaged endothelial cells are able to stimulate neurogenesis, oligodendrogenesis, and the angiogenesis mediated by progenitor endothelial cells. Through their close relationship with pericytes, they play a key role in replenishing the blood brain barrier, in the synthesis of the extracellular matrix, and in angiogenesis [35, 77–81].

Ischemic injury to the white matter is an important source of free radicals and oxidative stress damage. Here, oligodendrocytes will provide essential substrates for axonal recovery, and together with endothelial cells, will contribute to angiogenesis and oligodendrogenesis [82, 83].

Microglia has contradictory functions, which may either be cytoprotective or cytotoxic, during cerebral ischemia, as they release pro-inflammatory or anti-inflammatory molecules, attenuate or stimulate neurogenesis, and inhibit or promote phagocytosis. The reasons for this phenotypic heterogeneity are not yet well-understood [84, 85].

The cellular and extracellular behaviours of the neurovascular union and the molecular expression changes during cerebral ischemia (Fig. 3) sometimes have a dual nature. The activation of NMDA receptors, which are responsible the early excitotoxicity peak, is essential for neuronal plasticity [15, 16]. Oxidative stress is involved in cellular membrane destruction, but is essential for mediating the release of trophic factors and oligodendrogenesis [86]. Perhaps the paradigm of heterogeneous molecular reactions lies within inflammatory molecules. Cerebral ischemia triggers an inflammatory response, which is mostly responsible for cellular necrosis and the breakdown of the blood brain barrier. However, this response is also crucial for cellular repair and the regeneration of the affected parenchyma. Recently, several studies have demonstrated that persistence of the inflammatory response may be responsible for neurodegenerative processes after stroke [19, 87–93].

Consequently, the dynamic and heterogenic characteristics of ischemic process are the main responsible of the development of the personalized medicine. Future therapies must be designed taking into account different parameters as cellular or process goal, time and affected regions.

## Personalized neuroprotection

There are many variables that have led to the failure of neuroprotection as an alternative therapy in nervous system diseases, particularly in stroke. In this work, we have discussed the complexity of cellular and molecular mechanisms during cerebral ischemia. This complexity underlies the difficulty of achieving universal neuroprotection. To overcome this obstacle, several approaches have been proposed. These include the use of combined treatments with additive or synergistic benefits or treatments with pleiotropic effects [10]. However, these options have not yet been effective.

Given the urgent need to improve neuroprotective approaches, it seems necessary to develop customized strategies that facilitate access to specific targets at the right times. This ambitious goal requires the development of biomarkers to quickly evaluate the stage of molecular mechanisms and drug-carrying devices that can cross the intact blood brain barrier and reach the affected areas of the brain parenchyma.

Neuroscience research in the last decade has been focused on the development of biomarkers for diagnosis, treatment, and prognosis after stroke. However, few of these biomarkers, if any, have been applied in clinical settings. In addition, only a few clinical trials have included the use of biomarkers [94]. Nevertheless, all of these studies and their results have contributed to a better understanding of the pathophysiology of brain ischemia.

One of the main factors reducing the translation of biomarkers of excitotoxicity, oxidative stress, inflammation, endothelial dysfunction, apoptosis, etc. is that they are time-dependent. One limitation of the use of these biomarkers is that they may reflect the effects of the damage rather than the mechanisms responsible for the damage at the time of their determination [95]. Another limitation is the low sensitivity and low specificity of the biomarkers used so far. Neurons, astrocytes, microglia, oligodendrocytes, endothelial cells, and pericytes all secrete the same biomarkers, even though they may have antagonistic functions depending on their energetic availability or their particular localization within the damaged or healthy brain parenchyma [96]. Immediately after ischemia, excitotoxicity reaches a maximum at the core of the lesion, which is a region of reduced therapeutic interest. However, excitotoxicity is delayed in the penumbra [97, 98]. In perfused tissue, there are high levels of oxidative stress, which are associated with better outcomes. In addition, identical inflammatory biomarkers in this region may have opposite effects depending on timing, localization, cell type, and the hemodynamic situation [87, 89, 99–101]. On the other hand, not all these biomarkers are secreted to the blood stream, some of them are only expressed on the surface of affected cells being unable to perform a direct detection trough non-invasive techniques like blood extraction.

Since stroke is a heterogeneous and time-dependent disease, the identification of a unique biomarker that can reflect all of the complicated pathophysiologic processes is difficult. The combination of several biomarkers and their evolution in time would probably provide precise an specific information regarding what is happening in the affected region during and after ischemia [102]. To achieve this objective and to move further toward personalized neuroprotection, there is a need for new clinical [103] and preclinical [104] perspectives that can lead to the development of useful and accurate biomarkers. The detection of serum or cerebrospinal fluid biomarkers are a well-established method for diagnostic and to check the evolution of many diseases such as ischemic stroke or Alzheimer [68, 102–106], but is limited to soluble biomarkers in blood suspension that can be extracted from the patients multiple times, that hardly reduces the diagnostic of many diseases that doesn’t meet these requirements like the extracellular expression of an specific protein related to an specific pathology that is not fully excreted to blood stream and consequently undetectable. In this way, nanoparticles are a powerful tool for both treatment and diagnostic [107]. A nanoparticle composed by a contrast agent/radiolabel and functionalised with antibodies against an specific target in cell surfaces allowing to assess the evolution/detection in a non-invasive way. Also, if this is combined with drug encapsulation, allows an specific and local treatment that increases drug efficiency and dose reduction limiting secondary effects. A dual methodology combining nanotechnology and common biomarkers could highly improve diagnostic and treatment in neurological disease, which represents the basis of the concept of personalized medicine.

Another element that may lead to misleading interpretations of biomarkers during cerebral ischemia is the BBB. Normally, the main function of the BBB is to protect the central nervous system from the entrance of drugs. However, this role may be reversed, and the BBB may modulate biomarker release from the brain to the general circulation [107–109].

The time-evolution of blood–brain-barrier permeability after acute ischemic stroke is a controversial and not well understood issue in humans. Briefly, although research works present controversy about the permeability window (from 1, 4, 24 and 48 h from the onset of the lesion up to 1 week), all studies agree that during the acute phase of stroke, a transitory breakdown of the BBB occurs, which facilitates the systemic infiltration to the ischemic surrounded area [110]. The concentrations of these molecules depend on their cerebral concentration but also on the degree of BBB dysfunction. Thus, the optimal combination of molecular [111–113] and neuroimaging [114] biomarkers would help in elucidating the integrity of the BBB and therefore to understanding changes in the concentrations of biomarkers in plasma.

It should also be noted that one of the biggest challenges in the study of neuroprotection in stroke is the translation of animal studies to human trials. Different treatments that produce positive results in rodents have failed to provide significant benefit in clinical trials. Detailed discussion of animal stroke models and their application in clinical studies can be found in several research works [115–118]. Briefly, recreating all features of human stroke in an animal model is not feasible due to complexity of the disorder. Some of the factors that we should be considered for the personalized neuroprotection therapy development include age and medication use history of the patient, infarct size, location and collateral circulation. In this line, choosing an appropriate animal stroke model and optimizing the study design increase the translation from animal research to clinical applications.

Last, research and clinical works indicate that personalized neuroprotection will be in the next years a reality as a consequence of a process of adaptation to the nanotechnology, which has proven to be an innovative approach for diagnostic and drug delivery therapies.

## Nanotechnology for the diagnosis of different developmental stages of ischemic stroke

A key for the development of an efficient neuroprotective therapy is the early identification of specific brain regions that can be treated using a particular approach. In the past several centuries, and up to the 1970s, the diagnosis of stroke was based exclusively on clinical symptoms. In fact, most of the extraordinary progress that has been made in recent years is due to our exceptional ability to visualize the brain. Neuroimaging has enabled the accurate determination of structural and functional alterations in the nervous system. However, conventional techniques often have several restrictions that limit the information provided. These include reduced biomarker sensitivity and specificity for identifying and classifying disease, short half-lives of contrast agents after systemic administration, and the physical restrictions of the BBB [119]. Molecular magnetic resonance imaging (MRI) techniques with lower anatomic resolutions have proven more effective than structural imaging techniques in the study of nervous system function [120].

The use of neuroimaging tools combined with nanoparticles has improved the resolution of the current diagnostic procedures. Nevertheless, recent advances in the synthesis and functionalization of these nanoparticles are now leading to the development of much more specific diagnostic approaches for the nervous system [119].

Iron oxide nanoparticles have been extensively investigated as magnetic resonance contrast agents for last two decades, and several types of these particles, such as *ferumoxytol*, have been approved by the Food and Drug Administration for use in clinical practice [2]. In particular, the use of multifunctional superparamagnetic iron oxide nanoparticles (SPIONs) has gained growing interest for use in clinical applications. In general, SPIONs have an iron oxide core (approximately 10 nm) coated with biocompatible polymers and can be classified as cross-linked iron oxide nanoparticles (CLIONs, 50–180 nm), ultra-small superparamagnetic iron oxide nanoparticles (USPIONs, 10–50 nm), and very-small superparamagnetic iron oxide nanoparticles (VSPIONs, <10 nm). All of these SPIONs have the potential to cross the BBB due to them special properties, long half-lives in blood circulation, and reduced toxicity, besides this, interactions with plasma proteins (formation of the protein corona) and other blood components could play a pivotal role crossing the BBB by increasing the size and modifying the charge. Because of this interaction studies are mandatory in order to make sure particles preserve their functionalization properties in biological fluids [121, 122]. SPIONs are eventually eliminated by macrophages, unless they are modified to increase half-lives in blood circulation and due to their inherent magnetic properties, can be visualized in T2- and T2*-weighted MR images [123].

**Fig. 4:**
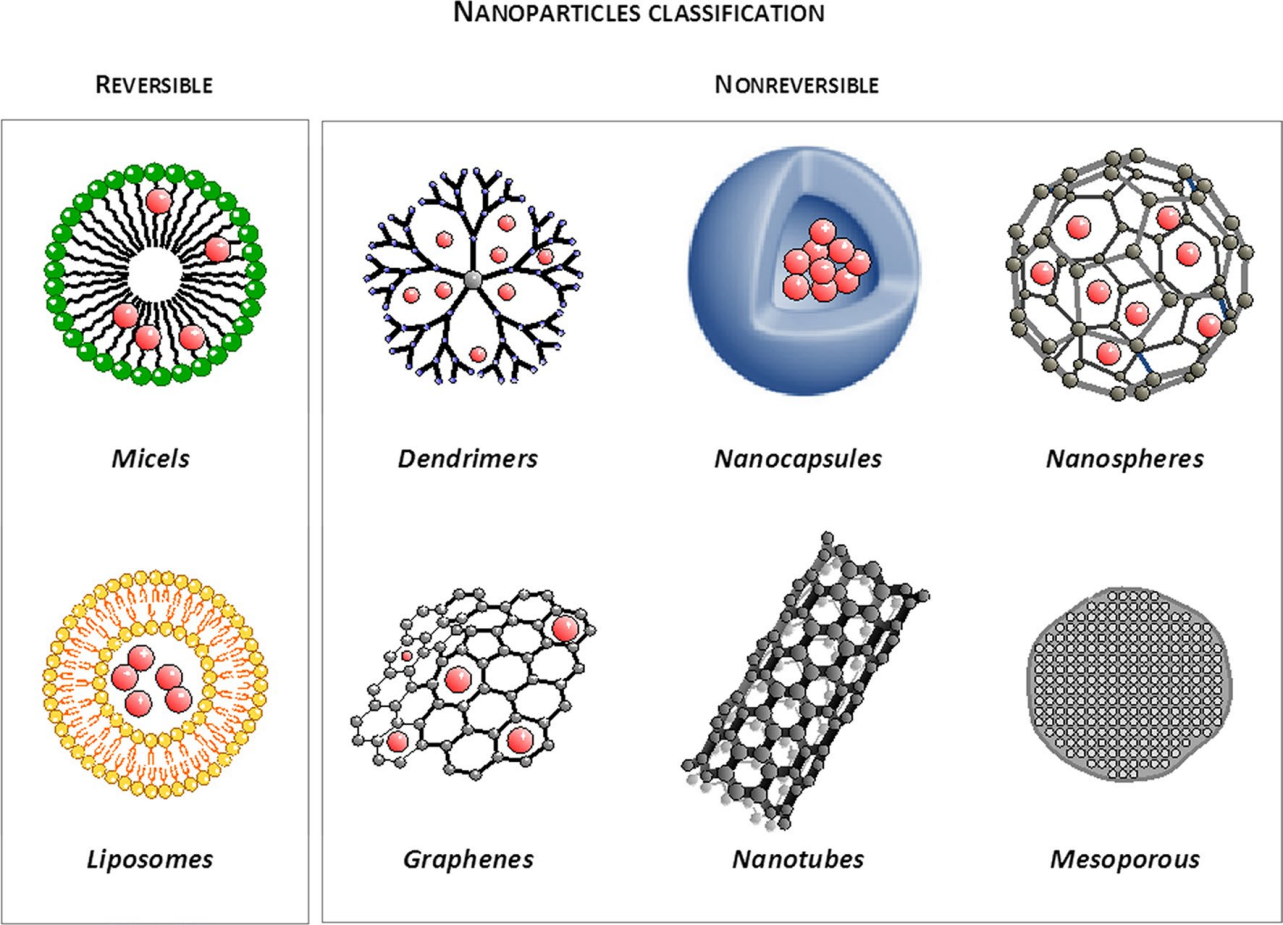
Schematic representation of different types of nanoparticles used for diagnosis and as drug delivery systems

Multifunctional modifications of magnetic nanoparticles or their bioconjugation to antibodies, peptides, aptamers, or other specific molecules, will allow for nanoparticle vectorization and their accumulation in particular regions. This technology enables the identification of specific regions in the brain parenchyma affected by particular pathophysiological or pathogenic mechanisms. This may then be used to focus drug delivery within the optimal therapeutic window [124]. This hypothesis is now under investigation (Fig. 4).

**Fig. 5:**
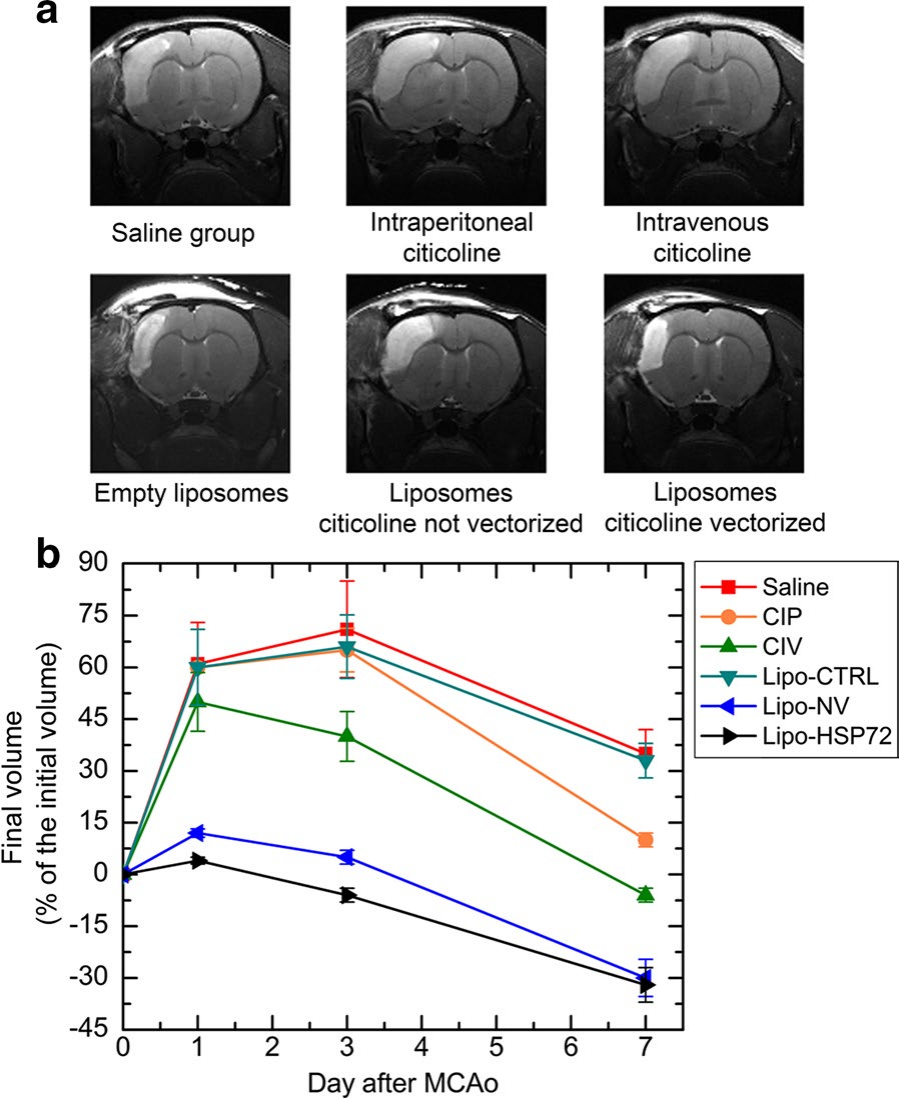
Volume of the residual lesion after transient middle cerebral artery occlusion (tMCAo) (1 h) using citicoline alone, citicoline encapsulated in non-vectored liposomes, or citicoline vectored with antibodies to the heat shock protein-72 expressed in the area of the ischemic penumbra (Adapted from [129] by permission of *Theranostics*)

Iron oxide nanoparticles have already been used as selective markers for P-selectin and other adhesion molecules and have allowed the detection of early inflammatory responses in animal models of brain ischemia [125, 126]. For instance, liposomes loaded with gadolinium and labelled with antibodies against heat shock protein-72, which is a chaperon that is expressed at high concentrations in the ischemic penumbra [127], have allowed the MRI visualization of the peri-infarct region a few hours after the occlusion of the middle cerebral artery [128, 129] (Fig. 5). The development of future multifunctional nanoplatforms will open the doors for more sensitive and specific biomarkers that can be used for a more accurate diagnosis and eventually the identification of potential therapeutic targets [130]. However, parameters such as biodistribution, pharmacokinetics, and toxicity of these nanoplatforms must be addressed first. In this sense, novel nanosystems will have to be non-toxic, biocompatible, biodegradable, and easily detectable at reduced concentrations. Therefore, it is important to note that currently there are laying the foundation of future personalized medicine for the diagnosis and treatment of ischemic stroke, however we consider that combining time-accurate biomarkers and adequate drug delivery systems able to cross the BBB are the main imminent needs to improve.

## Nanotechnology for the treatment of ischemic stroke and for cell tracking

In addition to new safe and efficient diagnostic tools, *nanoneuromedicine* may be used to develop new therapeutic approaches for nervous system diseases that have so far been untreatable [124].

Nanotechnology operates at the same length scale as natural processes involving viruses, cells, and bacteria, and allows us to interact with these biological entities in a very specific and localized manner. This has opened the door for new approaches in drug administration. For example, drugs can be dissolved, mingled, encapsulated, or linked to nanoparticles, and then delivered and monitored within a specific region of the body.

Nanoparticles with sizes ranging from 1 to 100 nm are capable of interacting with biological systems at a molecular level. They are even able to encapsulate or establish stable complexes with a variety of drugs. Drug/molecule nanoencapsulation increases the efficacy, specificity, and tolerance for drugs (due to the reduced amount of drugs required and that nanoparticles are able to modify pharmacokinetic properties of treatments). Therefore, nanoencapsulation further increases the therapeutic benefits of drugs. In addition, drug encapsulation can be used to delay or even stop drug degradation during interactions with the biological environment. Drug encapsulation may also facilitate the absorption of drugs and increase their cellular penetration [131].

Multiple nanoparticles have been tested for drug delivery. Nanocarriers can be classified into two groups, reversible and non-reversible carriers [132]. Liposomes and micelles are the best known reversible nanocarriers and their supramolecular complexes are generated on the basis of non-covalent intermolecular interactions. However, changes in environmental conditions usually result in disaggregation of the molecular units that form the particle, making them less suitable for preparations of stable commercial products used by the pharmaceutical industry. On the other hand, the broad family of non-reversible nanoparticles, including nanospheres, nanocapsules, dendrimers, metal and magnetic nanostructures, carbon nanotubes, and mesoporous materials [133–135] have strong molecular interactions, which allow them to have a high degree of chemical stability. This in turn facilitates their manufacturing for commercial purposes [12] (Fig. 4).

Nanostructures have the potential to cross the BBB. Although the BBB is disrupted during the acute phase of stroke and transport across the BBB is not regulated during the subacute phase or the chronic stage of the disease, adequately coated nanoparticles are able to pierce the intact BBB by cell-mediated transcytosis mechanisms and perform brain-localized drug delivery [134, 136, 137].

How the nanostructure encapsulated drug, vectorized through the corresponding biomarker, could reach the ischemic territory, hence, provide diagnosis and/or treatment for ischemic stroke may seem insurmountable obstacle during the ischemic disease process. However, it is well known that cerebral collaterals are vascular redundancies in the cerebral circulation that can partially maintain blood flow to ischemic tissue when primary conduits are blocked [138]. Thus, after occlusion of a cerebral artery, anastomoses connecting the distal segments of the middle cerebral artery occlusion (MCAo) with distal branches of other cerebral arteries allow for partially maintained blood flow in the ischemic penumbra and delay or prevent cell death. Therefore, even without a complete arterial reperfusion, nanoparticles can potentially get the core or peripheral ischemic territory to achieve the designed proposed. In addition, the use of nanoparticles has been also combined with thrombolytic treatments [139] with the aim to improve the efficacy of arterial reperfusion and reduce the risk of bleeding. In this regard, the use of nanotechnology in combination with thrombolytic and neuroprotective treatments, for instance, may overcome the application of the nanotechnology on those stroke condition where the cerebral artery remains still occluded.

Nanostructures differ in sizes (ranging from nanometers to micrometers), shapes (from spherical, hemispherical, cylindrical, and even conical), surface and composition [140]. In the same line, to clinical application in ischemic stroke, we must take into account the main characteristics of the nanoparticles required to traverse the BBB, which are listed below [141, 142]: diameter less than 100 nm, be non-toxic, biodegradable and biocompatible, be stable in blood, have prolonged circulation time, not activate neutrophils, not lead to platelet aggregation, be BBB-targeted and controlled drug release. Several studies have demonstrated that these nanoparticles can be transported by axons. In addition, it has been shown that after nasal administration, iron oxide nanoparticles are found in the olfactory bulb, striatum, hippocampus, cerebral spine, cerebellum, and frontal cortex [143]. This may open the door for new administration routes.

Carbon nanotubes have a wide range of chemical, electrical, and mechanical properties, and have been previously reported to be powerful antioxidants [144] and promising scaffolds for tissue repair [134, 145, 146].

**Fig. 6:**
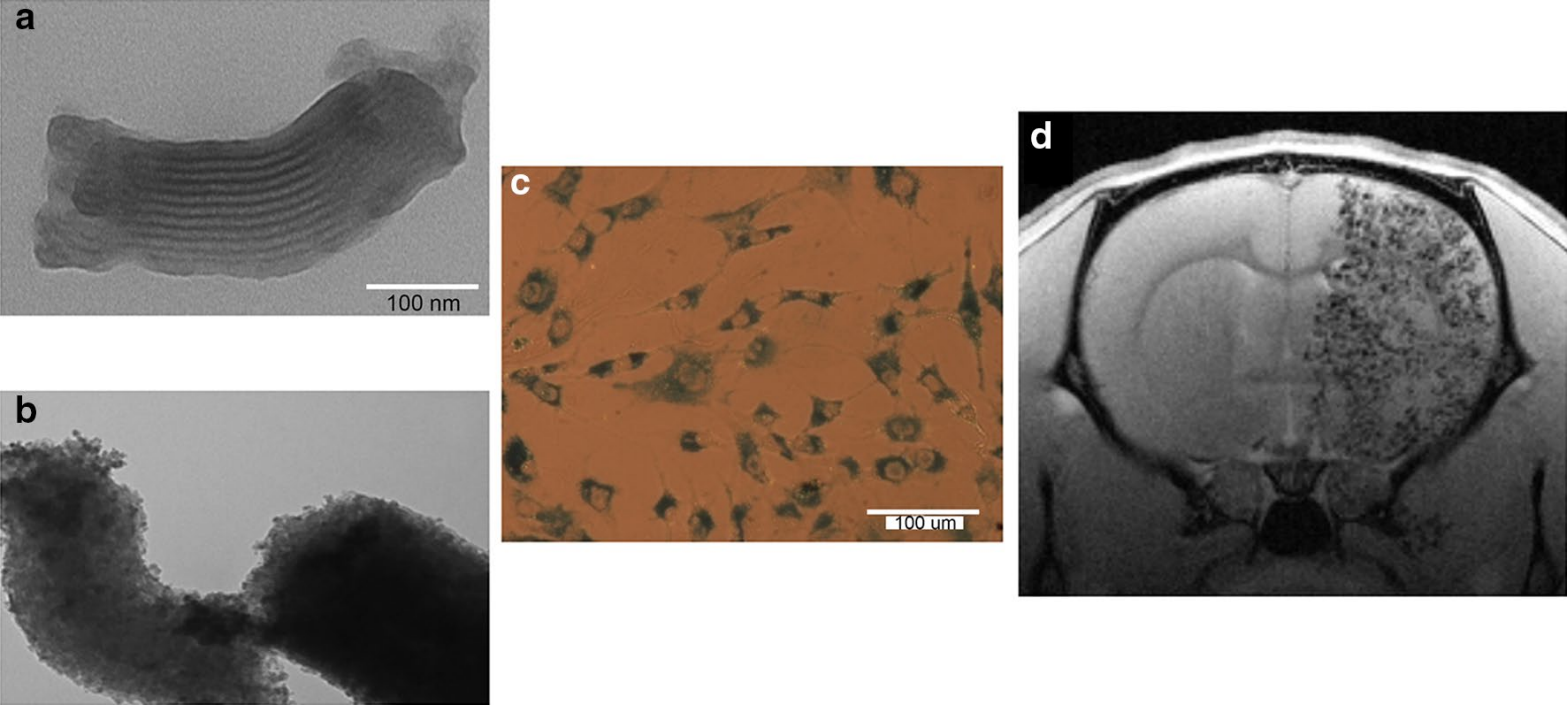
**a** Mesoporous nanostructures and **b** iron oxide-functionalised mesoporous nanoparticles. **c** In vitro tolerance. **d** Magnetic resonance T2*-weighted image of one brain slice of a Wistar rat after mesenchymal cell administration labelled with dextran-coated superparamagnetic nanoparticles (D-MNPs) (Adapted from [157] by permission of *Scientific Reports*)

In addition to different nanostructures based on thermoreversible polymers or liposomes designed to ensure enhanced drug delivery control, mesoporous nanoparticles have lately attracted intense attention due to their chemical versatility and textural properties. The highly interconnected and ordered pores of these materials allow them to store huge amounts of drugs and maintain their pharmacological properties for long periods after their administration. In addition, they offer a free surface for functionalization with specific tagging agents allowing for further vectorization functionality. Moreover, high biocompatibility, biodistribution, and excretion through the digestive system [147–150], as well as the ability to undergo cellular internalization by endocytosis without provoking cell alterations (Fig. 6), are additional properties that make these materials highly desirable for biomedical applications.

Another emergent therapeutic modality is stem cell therapeutics, which is designed to promote tissue repair after stroke. This therapeutic strategy has two aims: to re-establish the different cellular populations, and to restore neurological function. Nevertheless, recent studies have shown that the mechanisms of action of stem cells are still not well-understood, as these cells may be involved in neurotrophic factor secretion, immunomodulation, endogenous neurogenesis stimulation, or neovascularization [150]. Likewise, nanomedicine may be viewed as a necessary complement of cell-based therapies due to excellent biocompatibility, ease of cellular internalization, and in vivo cell tracking possibilities. Nanomedicine may be a potential drug delivery platform used to induce specific differentiation- and cell-oriented therapeutics [148, 151–154].

The next step in stem cell therapy research is the development of new in vivo multimodal, non-invasive, and sensitive procedures to study the biodistribution, survival, migration capacities, and proliferation of the administered cells.

The optimum nanoparticles for use as contrast agents must be detectable by MRI at reduced doses, should cross the BBB, must be non-toxic, and must have no effects on cellular viability, mobility, and differentiation, and preserve cellular functions [149, 155–157]. Unfortunately, cell tracking presents several problems. For instance, it is not possible to distinguish between internalized iron in healthy stem cells and the iron signal from apoptotic cells or even macrophages using MRI. An ideal nanoparticle would have an MRI signal that disappears upon cellular death [158].

Finally, multiple concerns will need to be overcome before engineered nanomaterials for targeted drug delivery can become a reality in everyday clinical practice. As we have previously mentioned, nanoparticles have different features with broad and diverse functionalities depending upon their application. Issues such as large-scale production, cost-effectiveness, the potential toxicity of new nanomaterials, time of release, dose, and route of administration (oral, intraarterial, intravenous) are hot topics in current state-of-the-art nanomedicine [159].

## Conclusions

Neuroprotection is still an important objective in stroke research. Despite all of the advances in endovascular-perfusion therapy, the practical application of these techniques will always be highly limited for most patients. However, neuroprotection has the potential to be more universal if we are able to identify sensitive and specific biomarkers that reflect the temporal profile and local distribution of the pathophysiological mechanisms of stroke. Nanotechnology is already contributing to the field of personalized neuroprotection and has allowed us to identify the mechanisms underlying cerebral damage and determine optimal therapeutic windows for stroke in order to prevent patients from exposure to irreversible damage. In summary, we consider that these new methodologies may improve the treatment of stroke in the next future with a precise diagnosis of spatio-temporal evolution of ischemic lesion; identification of specific regions, classify domains and accurately identify the therapeutic window. Future perspectives will bring combination therapy for neuroprotection with more than one drug, administration route, cell line or strategy as systemic and focal hypothermia. Treatments will be more direct, effective and specific, with fewer side effects, and consequently the potential beneficiary patients will be considerably increased. Multiple aspects of these new *players* in biomedicine should be considered in future further in vivo and in vitro studies in order to improve their applicability in clinical studies.
